# Effect of Darkness on Intrinsic Motivation for Undirected Singing in Bengalese Finch (*Lonchura striata Domestica*): A Comparative Study With Zebra Finch (*Taeniopygia guttata*)

**DOI:** 10.3389/fphys.2022.884404

**Published:** 2022-05-25

**Authors:** Yunbok Kim, Chihiro Mori, Satoshi Kojima

**Affiliations:** ^1^ Sensory and Motor Systems Research Group, Korea Brain Research Institute, Daegu, South Korea; ^2^ Department of Molecular Biology, Faculty of Pharmaceutical Sciences, Teikyo University, Tokyo, Japan

**Keywords:** motivation, birdsong, zebra finch, Bengalese finch, stress, vocal learning, darkness, voluntary behavior

## Abstract

The zebra finch (ZF) and the Bengalese finch (BF) are animal models that have been commonly used for neurobiological studies on vocal learning. Although they largely share the brain structure for vocal learning and production, BFs produce more complex and variable songs than ZFs, providing a great opportunity for comparative studies to understand how animals learn and control complex motor behaviors. Here, we performed a comparative study between the two species by focusing on intrinsic motivation for non-courtship singing (“undirected singing”), which is critical for the development and maintenance of song structure. A previous study has demonstrated that ZFs dramatically increase intrinsic motivation for undirected singing when singing is temporarily suppressed by a dark environment. We found that the same procedure in BFs induced the enhancement of intrinsic singing motivation to much smaller degrees than that in ZFs. Moreover, unlike ZFs that rarely sing in dark conditions, substantial portion of BFs exhibited frequent singing in darkness, implying that such “dark singing” may attenuate the enhancement of intrinsic singing motivation during dark periods. In addition, measurements of blood corticosterone levels in dark and light conditions provided evidence that although BFs have lower stress levels than ZFs in dark conditions, such lower stress levels in BFs are not the major factor responsible for their frequent dark singing. Our findings highlight behavioral and physiological differences in spontaneous singing behaviors of BFs and ZFs and provide new insights into the interactions between singing motivation, ambient light, and environmental stress.

## Introduction

**FIGURE 1:**
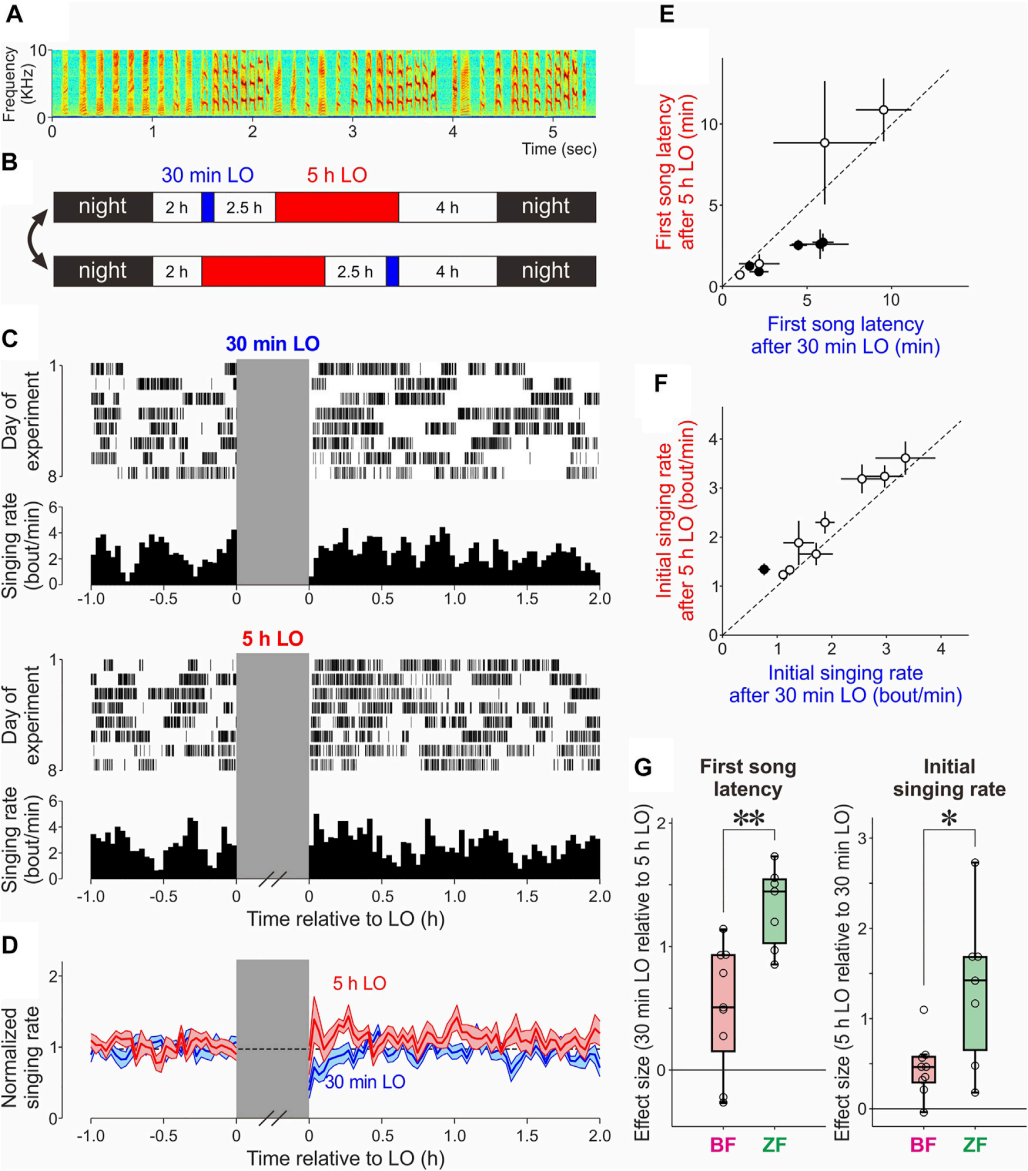
Effects of short (30 min) and long (5 h) lights-out (LO) on undirected singing in BFs and their comparison with those in ZFs. **(A)** Spectrogram showing one bout of undirected song in a representative BF. **(B)** Daily schedule of 30 min and 5 h LO periods. After a 2 h light period (white area) in the morning, a 30 min LO (blue area) and a 5 h LO (red area) were given with a 2.5 h intervening light period, followed by a 4 h light period. The order of the 30 min and 5 h LO was switched every 1–3 days. Each row indicates the schedule on one day. **(C)** Raster plot of song bouts produced before and after a 30 min LO (top) and 5 h LO (bottom) and corresponding singing rate histograms (bin size is 2 min) in a representative bird. **(D)** Time course of instantaneous singing rate before and after a 30 min (blue) and 5 h LO (red), normalized to the mean singing rate before LO (mean ± SEM, *n* = 9 birds). **(E)** The first song latencies (mean ± SEM) after a 5 h LO plotted against those after a 30 min LO (*n* = 9 birds). Filled circles indicate birds with statistical significance between a 5 h and 30 min LO data (*p <* 0.05). The dashed lines indicate unity. **(F)** Initial singing rates after a 5 h LO plotted against those after a 30 min LO (*n* = 9 birds). Conventions are as in E. **(G)** The effect sizes [Hedges’ g for the first song latency (left) and the initial singing rate (right) between a 30 min and 5 h LO in BFs and ZFs. *p < 0.05; **p < 0.01].

Songbirds have been widely used in behavioral and neurobiological studies because of their remarkable abilities of vocal learning. Bengalese finches (BFs) and zebra finches (ZFs), the two most commonly used songbird species in neurobiology, learn to sing by imitating the songs of other conspecifics during a critical period of development and share many aspects of neural circuits and mechanisms of song production and learning (Murphy et al., 2017). However, despite these common features, BFs and ZFs show considerable differences in their song structure: ZFs produce songs with repetitions of a highly stereotyped sequence of song notes or “syllables,” whereas BFs produce much longer songs with highly variable syllable sequencing (Figure 1A) (Okanoya, 2004) with real-time control using auditory feedback (Okanoya and Yamaguchi, 1997). Such differences in song structure between BFs and ZFs despite the similarities in their brain structure make excellent models for comparative studies to understand how animals learn to produce and control complex motor behaviors.

In both BFs and ZFs, males spontaneously produce more than hundred renditions of songs a day throughout their life even when they are isolated from other individuals (Dunn and Zann, 1996; Derégnaucourt et al., 2005; Tumer and Brainard, 2007). Such spontaneous singing in a solo context, referred to as “undirected singing,” is thought to serve, at least in part, as vocal practice by which birds develop and optimize song structure (Konishi, 1965; Lombardino and Nottebohm, 2000; Brainard and Doupe, 2001; Tumer and Brainard, 2007). Undirected songs are produced in the absence of any immediate external rewards and appear to be driven by intrinsic motivation to obtain internal reward (Riters and Stevenson, 2012), providing a unique opportunity to study the mechanisms of intrinsic motivation that drives the learning and production of complex motor skills.

We recently found in young adult ZFs that temporary suppression of undirected singing by turning off the ambient light enhances intrinsic motivation for undirected singing to an extent that depends on the duration of singing suppression (Kim et al., 2021): birds sing much sooner and more intensely after relatively long (5 or 10 h) periods of lights-out (LO) than after shorter LO periods. In the present study, we use the same procedure in BFs to examine the possible differences in the enhancement of intrinsic motivation for undirected singing between BFs and ZFs. Our results revealed substantial differences between the two species in LO-induced enhancement of singing motivation as well as singing behavior during LO periods. We also investigated the physiological mechanisms underlying such behavioral differences between the two species by measuring blood corticosterone (CORT) levels under both dark and light conditions.

## Materials and Methods

### Subjects

Subjects were adult male BFs [*Lonchura striata domestica*, 91–128 days post-hatching (dph)] and ZFs (*Taeniopygia guttata*, 87–119 dph). Birds were raised in our colony with their parents and siblings until ∼60 dph and then housed with other conspecifics until the experiments started. Care and treatment of animals were reviewed and approved by the Institutional Animal Care and Use Committee (IACUC) of the Korea Brain Research Institute. All experiments were performed following the relevant guidelines and regulations.

### Song Recording

Birds were housed individually in sound-attenuating chambers (MC-050, Muromachi Kikai) under a 14/10 h light/dark cycle throughout the experiments. Undirected songs were recorded as previously reported (Kim et al., 2021). Briefly, the output from a microphone (PRO35, Audio-Technica) positioned above the cage was amplified using a mixer (402-VLZ4, Mackie) and digitized via an audio interface (Octa-Capture UA-1010, Roland) at 44.1 kHz (16-bit). The recording was controlled by a custom-written song recording program (R. O. Tachibana of the University of Tokyo), which triggered recording if it detected four or five consecutive sound notes, each of which was defined based on the sound magnitude, sound duration, and intervening gap duration. Each recording ended when the silent period lasted longer than 0.5 s. Birds with sufficient singing rates (>300 song bouts per day) were used for further experiments (2/19 ZFs and 0/17 BFs were rejected because they did not satisfy this criterion). All ZF song data were originally collected for a previous study (Kim et al., 2021) and re-analyzed for comparison with the BF data.

### Temporary Singing Suppression and Song Analysis

To suppress undirected singing, the light in the sound-attenuating chambers was turned off using digital timers. The duration and schedule of lights-out (LO) periods varied depending on experimental paradigms (from 30 min to 10 h; see Results); individual birds received LO of the same duration 6–8 times. Singing behaviors following individual LO periods were quantified using two measures, the first song latency and initial singing rate (Kim et al. (2021)). The first song latency was measured as the time interval from the offset of a LO period to the onset of the first song recorded. We visually inspected the spectrograms of the sound files recorded after the LO periods to identify the first file that included at least one song motif. The initial singing rate was measured as the mean singing rate over a 30 min period starting at the onset of the first song produced after an LO period (the timing of the 30 min period varied across trials, depending on the first song latencies). To measure singing rates, we screened all sound files recorded during the periods of interest to exclude non-song files using a previously reported semi-automated method (Kim et al., 2021). Briefly, we sorted song files (files that included at least one full song motif) and other sound files by focusing on the temporal structure of two acoustic features: sound amplitude and Weiner entropy. We compared the trajectories of these features between a canonical song motif and all sound files recorded by calculating the cross-correlation function. Sound files that had relatively low correlation coefficients in both the amplitude envelope and envelope trajectory were then excluded from the analysis.

To quantify the degree of singing motivation enhancement caused by LO, we calculated the Hedges’ g effect sizes for both the first song latency and the initial singing rate between 30 min and 5 h LO or 30 min and 10 h LO. For each bird, the mean and SD of either the first song latency or the initial singing rate were computed across trials for both short (30 min) LO and long (5 h or 10 h) LO, and the effect size was calculated using the following formula, which includes a correction for small sample sizes (Hedges, 1981):

$$g=\frac{{\bar{x}}_{1}-{\bar{x}}_{2}}{SD^{*}}\times \;\left(\frac{N-3}{N-2.25}\right)\times \;\sqrt{\frac{N-2}{N},}$$

where ${\bar{x}}_{1}$ , ${\bar{x}}_{2}$ , $SD^{*},$ and *N* denote the mean of the data 1, the mean of the data 2, the pooled standard deviation, and the sum of the sample sizes (i.e., the number of trials) of the data 1 and 2, respectively. The $SD^{*}$ was computed as:

$$SD^{\text{∗}}=\sqrt{\frac{\left(n_{1}-1\right)SD_{1}^{2}+\left(n_{2}-1\right)SD_{2}^{2}}{n_{1}+n_{2}-2},}$$

where $n_{1},\;$ $n_{2}$ , *SD*_*1*_, *SD*_*2*_ denote the sample size of the data 1, the sample size of the data 2, the standard deviation of the data 1, and the standard deviation of the data 2, respectively. When calculating the effect sizes for the first song latency, which tends to be relatively long after a short LO in ZFs (Kim et al., 2021), the data with 30 min LO were assigned to the data 1 and the data with longer LO (5 or 10 h) were assigned to the data 2 so that the effect size of most ZFs become positive. For the same reason, when calculating the effect sizes for the initial singing rate, which tends to be relatively large after a long LO in ZFs, the data with longer LO (5 or 10 h) were assigned to the data 1 and the data with 30 min LO were assigned to the data 2.

### Corticosterone Assays

Blood CORT levels under both dark and light conditions were measured for each experimental bird. Before blood sampling, the birds were housed individually in sound-attenuating chambers for >3 days. On the day of blood sampling in dark conditions (“Dark day” in Figure 4A), 6 h LO was conducted in the middle of the day, and 50–200 µl of blood was collected 1 h before the end of the LO period by puncturing the brachial wing vein with a sterile needle (23G). On the day of blood sampling in light conditions (“Light day” in Figure 4A), blood was collected at the same time of the day, but no LO was given. To minimize the potential stress caused by the blood sampling operation, all blood samples were collected within 3 min after opening the sound-attenuating chambers to take the birds out of their cages. The blood samples were centrifuged at 3,500 rpm for 20 min at ∼20°C, and the sera were stored at −20°C until the assay. The dark and light days were repeated 3–5 times in an interleaved manner until a total of >100 µl of serum was collected under both dark and light conditions (2/14 BFs and 4/15 ZFs were rejected because we could not collect enough serum samples from them). CORT levels were measured using enzyme immunoassay kits (Cat. ADI-900-097, Enzo Life Sciences). All serum samples (1:10 dilution) and standards were analyzed according to the manufacturers’ instructions. The plates were read on an absorbance 96-plate reader (SpectraMax® M2, Molecular Devices). CORT levels were determined using 5-parameter logistic regression software of CORT standards ranging from 32 to 20,000 pg/ml.

### Statistical Analysis

To analyze the effects of LO on singing behavior, we compared first song latencies and initial singing rates between 30 min LO and 5 h LO for each bird using a Wilcoxon signed-rank test (*α* = 0.05), which was also used for the group data. We examined the effects of LO with four different durations on singing behavior using Friedman’s test. To compare the enhancement of intrinsic singing motivation between BFs and ZFs, Fisher’s exact test was used for the ratio of birds with statistical significance and a Mann–Whitney U test for the effect sizes (Hedges’ g). For the data of blood CORT levels, we used a Mann–Whitney U test to compare the data between BFs and ZFs and a Wilcoxon signed-rank test to compare the data between dark and light conditions. All statistical analyses were performed using the MATLAB software.

## Results

### Bengalese Finchs Exhibit Suppression-Dependent Enhancement of Undirected Singing Motivation to a Smaller Degree Than Zebra Finchs

Our previous study in ZFs demonstrated that temporary suppression of undirected singing by turning off the ambient light enhances intrinsic motivation for undirected singing to an extent that depends on the duration of singing suppression: ZFs sing much sooner and more intensely after relatively long (5 h) periods of lights-out (LO) than after shorter (30 min) LO periods (Kim et al., 2021). Using the same experimental procedures, we examined whether BFs exhibit such differences in post-LO singing behavior between 5 h LO and 30 min LO to degrees similar to those observed in ZFs. As in the previous study in ZFs (Kim et al., 2021), young adult male BFs (91–128 dph) were subjected to both 30 min and 5 h LO periods on each day (they were separated by a 2.5 h light period and their order was switched every 1–3 days; Figure 1B). We found that BFs exhibited differences in post-LO singing behavior between 5 h LO and 30 min LO that were much less clear than those observed in ZFs. BFs appeared to recover singing rates to the pre-LO levels faster after 5 h LO than after 30 min LO on average (Figures 1C,D), and the latency to the first song after LO periods (“first song latency”) was significantly smaller after 5 h LO than after 30 min LO in more than half of birds (5/9 birds, Wilcoxon signed-rank test, *α* = 0.05 was used for each bird) (Figure 1E). As a group, however, no significant difference in first song latency was observed between 30 min LO and 5 h LO (Figure 1E, *n* = 9 birds, *p* = 0.25, Wilcoxon signed-rank test), sharply contrasting with the striking and significant difference in the same measure in ZFs (Kim et al., 2021). To compare the degrees of singing motivation enhancement between BFs and ZFs more directly, we re-analyzed the ZF song data that were originally collected for our previous study (Kim et al., 2021) to calculate the effect sizes (Hedges’ g; see Methods) of first song latencies between 30 min LO and 5 h LO, and compared them with the effect sizes of BF data. We found that the effect sizes were significantly smaller in BFs than in ZFs (Figure 1G, left, *n* = 9 BFs and 7 ZFs, *p* = 0.0021, Mann–Whitney U test). Moreover, although the initial singing rate (see Methods), another measure of singing motivation used in our previous study (Kim et al., 2021), was significantly greater after 5 h LO compared to that after 30 min LO in BFs (Figure 1F, *p* = 0.0078, *n* = 9 birds) as seen in ZFs (Kim et al., 2021), their effect sizes were significantly smaller than those in ZFs (Figure 1G right, *n* = 9 BFs and 7 ZFs, *p* = 0.031). In addition, the ratio of birds with a statistical difference in initial singing rate between 30 min and 5 h LO was significantly lower in BFs than in ZFs (1/9 BFs vs. 5/7 ZFs, *p* = 0.035, Fisher’s exact test); no significant difference was observed for first song latency (5/9 BFs vs. 6/7 ZFs, *p* = 0.31). Taken together, these results indicate that LO-dependent enhancement of intrinsic singing motivation is much smaller in BFs than in ZFs.

### Effects of Lights-Out With Four Different Durations on Singing Motivation in Bengalese Finchs and Their Comparison With Those in Zebra Finchs

**FIGURE 2:**
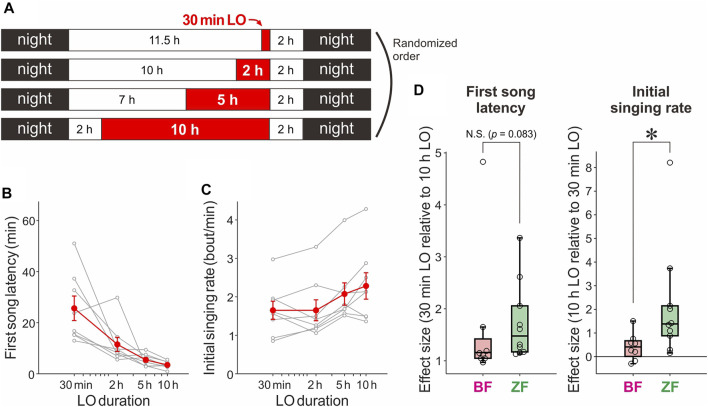
Effects of LO with 4 different durations (30 min, 2, 5, and 10 h) on singing motivation in BFs and their comparison with those in ZFs. **(A)** Daily schedule of LO periods (red areas) with four different durations. On each day, a single LO period with one of the four different durations was given with the offset at 2 h before night; the onset was varied depending on the LO duration. Birds received LO periods with four different durations in a randomized order. **(B)** First song latencies plotted against LO durations in BFs (*n* = 8 birds). Gray lines indicate data for individual birds and red lines represent mean ± SEM across all birds. **(C)** Initial singing rates plotted against LO duration in BFs (*n* = 8 birds). Conventions are same as in B. **(D)** The effect sizes for the first song latency (left) and the initial singing rate (right) between a 30 min and 10 h LO in BFs and ZFs *p < 0.05.

Kim et al. (2021) also characterized the suppression-dependent enhancement of singing motivation in ZFs using LO periods with four different durations (30 min, 2, 5, and 10 h). We used the same procedure in BFs and compared the results with those obtained in ZFs. On each experimental day, birds received a single LO period with one of the four durations; all LO periods ended 2 h before nighttime regardless of the duration to exclude possible circadian effects (Figure 2A). Consistent with the results of 30 min and 5 h LO shown in Figure 1, BFs exhibited enhancement of singing motivation depending on the duration of LO periods to a smaller degree than ZFs. Although the first song latencies monotonically decreased as the LO duration increased (Figure 2B, *n* = 8 birds; *p =* 0.00036, Friedman’s test) in a manner similar to those in ZFs (Kim et al., 2021), the effect sizes (Hedges’ g) calculated between the 30 min LO and 10 h LO data tended to be smaller in BFs than in ZFs (Figure 2D, left, *n* = 8 BFs and 10 ZFs, *p* = 0.083, Mann–Whitney U test; *p* = 0.014 after removing an apparent outlier in BF data). Moreover, unlike ZFs, which exhibit significant increases in initial singing rates depending on LO duration (Kim et al., 2021), BFs did not show significant increases in initial singing rates (Figure 2C, *n* = 8 birds; *p =* 0.054). Consistent with this, the effect sizes of the initial singing rate between the 30 min LO and 10 h LO data in BFs were significantly smaller than those in ZFs (Figure 2D, right, *n* = 8 BFs and 10 ZFs, *p* = 0.021).

### Bengalese Finchs Produce Undirected Songs More Frequently Than Zebra Finchs During Lights-Out Periods

**FIGURE 3:**
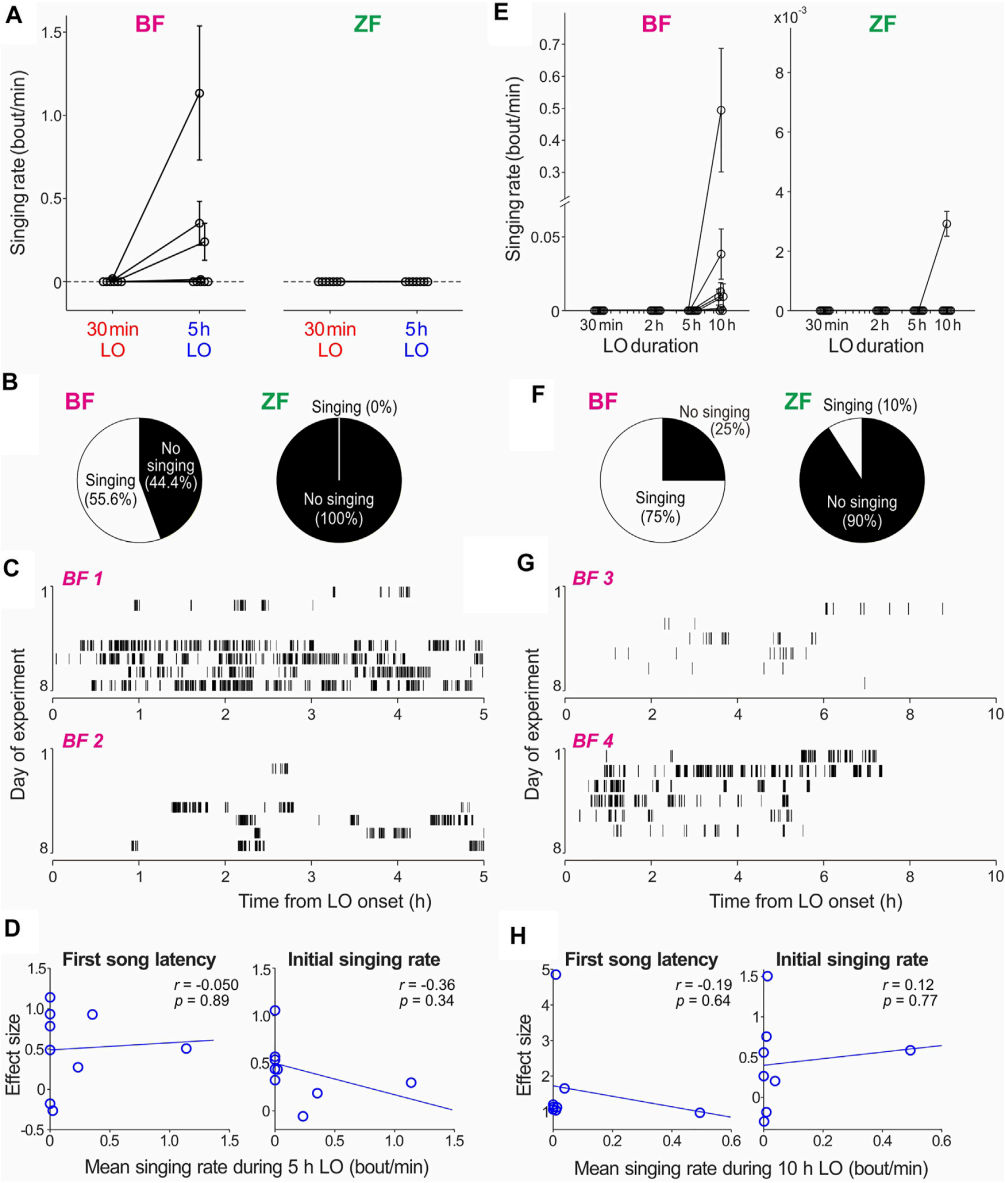
Comparison of BF and ZF singing behavior during LO periods. **(A)** Mean singing rates during 30 min and 5 h LO periods in BFs and ZFs (*n* = 9 BFs and 7 ZFs). **(B)** Percentage of BFs and ZFs that sung during 5 h LO periods. **(C)** Raster plots of song bouts produced during a 5 h LO in 2 representative BFs. **(D)** For all BFs examined, the effect sizes of first song latency (left) and initial singing rate (right) between the 30 min and 5 h LO are plotted against mean singing rate during 5 h LO periods. **(E)** Mean singing rates during LO periods with four different durations in BFs and ZFs (*n* = 8 BFs and 10 ZFs). **(F)** Percentage of BFs and ZFs that sung during 10 h LO periods. **(G)** Raster plots of song bouts produced during a 10 h LO period in two representative BFs. **(H)** Effect sizes of first song latency (left) and initial singing rate (right) between a 30 min and 10 h LO, plotted against mean singing rate during a 10 h LO.

To investigate the possible reasons for the difference in LO-dependent enhancement of singing motivation between BFs and ZFs, we examined singing activity during LO periods (“dark singing”) in both species. Interestingly, we found that a subset of BFs exhibited dark singing during relatively long LO periods: In the BFs that received the 30 min/5 h LO schedule shown in Figure 1B, 55.6% (5 out of 9 birds) produced at least one bout of undirected song during 5 h LO periods and one of those 5 birds also sung during 30 min LO periods (Figures 3A–C). Moreover, in the BFs that received the four different LO durations shown in Figure 2A, 75.0% (6 out of 8 birds) exhibited dark singing during 10 h LO periods, but no birds sang during any other LO periods (Figures 3E–G). Mean rates of dark singing during LO periods were highly variable across birds, ranging from 0 to 1.14 (mean = 0.19) bout/min for the 5 h LO and 0 to 0.49 (mean = 0.070) bout/min for the 10 h LO. In sharp contrast with BFs, almost none of the examined ZFs exhibited dark singing during LO periods. In the experiments with the 30 min/5 h LO schedule, none of the ZFs sung during any LO period (*n* = 7 birds; Figures 3A,B). Furthermore, in the experiments with four different LO durations, only 1 out of 10 ZFs (10%) sung during 10 h LO periods (Figures 3E,F), and the singing rate was relatively low (0.0029 bouts/min) compared to those of BFs. Thus, darkness almost completely suppresses undirected singing in ZFs, but much less strongly in BFs, revealing a striking difference in singing behavior between these species. This differential inhibitory effect of darkness on undirected singing may contribute to the difference in the LO-dependent enhancement of intrinsic singing motivation.

Our results so far in the current study and our previous study (Kim et al., 2021) demonstrate that longer and almost complete suppression of undirected singing causes greater enhancement of intrinsic singing motivation in ZFs whereas many BFs exhibited incomplete singing suppression and smaller degrees of singing motivation enhancement compared to ZFs. Given these findings, we wondered whether the frequency of dark singing (i.e., incompleteness of singing suppression) and the degrees of singing motivation enhancement are negatively correlated. More specifically, we assumed that BFs producing more frequent dark singing exhibit a smaller degree of LO-dependent enhancement of singing motivation, and vice versa. To test this possibility, we examined the relationships between the effect sizes of the LO-dependent enhancement of singing motivation (Figures 1G, 2D) and the amount of dark singing (Figures 3A,E) in BFs. However, we did not find any significant correlations between them either when the effect sizes were calculated from the first song latencies or the initial singing rates (Figure 3D for 5 h LO; Figure 3H for 10 h LO).

### Blood Corticosterone Levels Are Higher in Bengalese Finchs Than in Zebra Finchs

**FIGURE 4:**
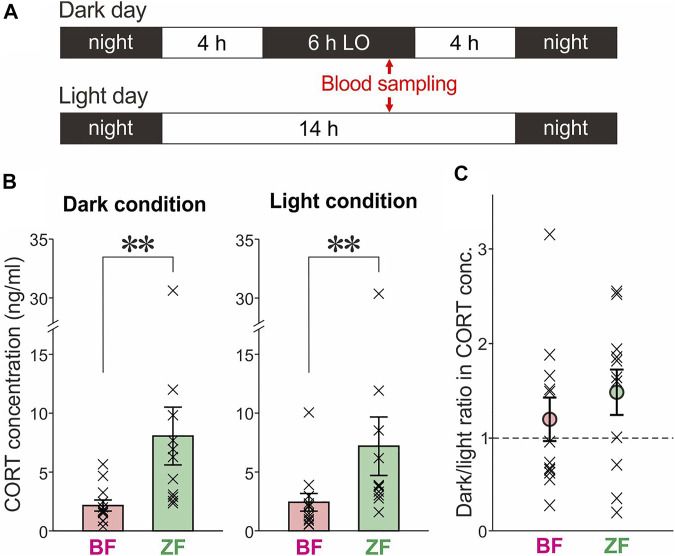
Comparison of blood CORT levels in dark and light conditions between BFs and ZFs. **(A)** Daily schedules of blood sampling with and without LO. **(B)** Blood CORT levels in BFs and ZFs under dark (left) and light (right) conditions. BFs showed significantly lower CORT levels than ZFs in both conditions (**p < 0.01). **(C)** Dark/light ratios in CORT levels were not significantly different from 1 either in BFs (*p* = 0.73) or in ZFs (*p* = 0.32). No significant difference was found between BFs and ZFs (*p* = 0.28).

The differential inhibitory effects of darkness on undirected singing between BFs and ZFs suggest that darkness affects the internal physiological state that regulates song initiation differently between the two species. One possible mechanism is that darkness differentially induces stress responses in these species. As singing in the dark generally increases the risk of predation (Lima, 2009), reducing the singing rate in the dark could be a stress reaction to cope with predation. BFs have been shown to exhibit lower degrees of anti-predator stress reactions induced by physical restraint than ZFs and BF’s wild ancestor, the white-rumped munia (WRM) (Suzuki et al., 2013; Suzuki et al., 2014), raising the possibility that more frequent dark singing in BFs than in ZFs is attributable to lower degrees of stress responses to darkness. To test this possibility, we measured the blood concentration of corticosterone (CORT), the main stress hormone in birds, in both dark and light conditions (Figure 4A) and compared them between BFs and ZFs. Previous studies have demonstrated that environmental stresses, which include the risk of predation, induce CORT release (Scheuerlein et al., 2001; Clinchy et al., 2004), and ZFs with higher CORT levels produce fewer songs (Wada et al., 2008). Therefore, if the more frequent dark singing of BFs is attributable to lower degree of stress responses to darkness, BF CORT levels in dark conditions must be lower than those of ZFs, and darkness would increase CORT levels to a smaller degree in BFs than in ZFs.

We found that blood CORT levels in dark conditions were significantly lower in BFs than in ZFs (Figure 4B, left; *n* = 12 BFs and 11 ZFs, *p* = 0.0019, Mann–Whitney U test). These results are consistent with the idea that the more frequent dark singing in BFs is attributable to the lower degrees of stress reactions caused by darkness. However, lower CORT levels in BFs than in ZFs were observed even under light conditions (Figure 4B, right; *p* = 0.0056). Moreover, the dark/light ratios in CORT levels were not significantly different from 1 (Figure 4C; *p* = 0.73 for BFs and 0.32 for ZFs, Wilcoxon signed-rank test), and there was no significant difference between BFs and ZFs (Figure 4C; *p* = 0.28). These results indicate that BFs have lower stress levels than ZFs regardless of whether they are in dark or light conditions, suggesting that stress responses are not a major factor suppressing undirected singing in dark conditions. Thus, frequent dark singing in BFs is unlikely to directly result from their lower degree of stress responses to darkness.

## Discussion

In the present study, we highlighted important differences in singing behavior between BFs and ZFs, the two most commonly used songbirds in neurobiological studies of vocal learning and communication. We found that the LO-dependent enhancement of intrinsic motivation for undirected singing was much smaller in BFs than in ZFs. Also, in contrast with almost no dark singing in ZFs during LO periods, a substantial portion of BFs exhibited frequent dark singing during relatively long LO periods, providing a plausible explanation for the smaller degree of LO-induced enhancement of intrinsic singing motivation in BFs. Moreover, although blood CORT levels were significantly lower in BFs than in ZFs in both light and dark conditions, no significant differences were observed between the conditions in either species. These comparative data in BFs and ZFs demonstrate that darkness differentially influences intrinsic motivation and production of undirected song between these species, and highlight the difficulty in manipulating undirected song production and motivation using darkness in BFs. Our results also provide new insights into the interactions between song production, intrinsic singing motivation, ambient light, and environmental stress.

Both BFs and ZFs have been extensively used to investigate the behavioral and neural mechanisms of vocal learning and communication. For example, a commonly used experimental manipulation to induce adaptive changes in the acoustic structure of adult song elements was first reported in BFs (Tumer and Brainard, 2007) and the neural mechanisms of such song changes have been intensively studied in both BFs and ZFs (Andalman and Fee, 2009; Charlesworth et al., 2012; Gadagkar et al., 2016; Hisey et al., 2018; Xiao et al., 2018; Tachibana et al., 2022). Because of the vocal learning ability and underlying neural circuits largely shared between these species, most studies have used them with little attention to species differences, although a limited number of studies took advantage of variable syllable sequencing in BF songs (Okanoya, 2004; Warren et al., 2012; Lipkind et al., 2013; Polomova et al., 2019; Veit et al., 2021). The current study systematically compared the behavioral properties of singing between the two species and investigated the underlying mechanisms, revealing previously unknown differences between them. Given the widespread use of both BFs and ZFs in neurobiological studies of vocal learning and communication, our findings, as well as other comparative studies in those species, including our recent study (Tachibana et al., 2022), broaden our understanding of the behavioral and neural mechanisms underlying learning and production of complex motor behaviors.

BF is a domesticated strain of the wild ancestor WRM, and a growing body of research suggests that in the course of the domestication process, BFs have acquired behavioral, anatomical, and neuroendocrine changes, including reduced sensitivity to environmental stresses (Suzuki et al., 2014; Suzuki et al., 2021; Suzuki and Okanoya, 2021; Tobari et al., 2022). In particular, BFs exhibit reduced levels of fearfulness related to coping with predation (Suzuki et al., 2013) and lower fecal CORT levels than WRM (Suzuki et al., 2012). We hypothesized that such reduced sensitivity to predation-related environmental stresses in BFs is a crucial factor underlying their frequent dark singing, and that hypothesis was partially supported by our results: The lower CORT levels in BFs than in ZFs in dark conditions (Figure 4B) are consistent with the idea that frequent dark singing in BFs is attributable to their reduced sensitivity to environmental stresses caused by darkness. Because darkness generally increases predation risk (Lima, 2009), this hypothesis also predicted that CORT levels would be higher under dark conditions than under light conditions in both species, but we did not find such cross-condition differences in either species (Figure 4C). This implies that singing suppression by darkness is mainly mediated by a physiological process that does not critically involve stress responses. Although darkness-induced reduction in singing is presumably an adaptive behavior that has evolved to reduce predation risk at night, it may be an automatic response to darkness that normally occurs independently of environmental stresses. This notion is also supported by our results in ZFs showing that intrinsic singing motivation is enhanced while singing is suppressed by darkness, because strong environmental stresses such as predation risk are likely to reduce intrinsic singing motivation rather than enhance it. Taken together, our CORT results lead to the conclusion that the frequent dark singing in BFs is not directly attributable to their reduced sensitivity to environmental stresses, but rather may reflect adaptive behavioral changes that BFs have acquired through domestication in captive environments with low predation risk. Of course, we cannot rule out the possibility that our blood sampling operations substantially affected CORT levels under both dark and light conditions and attenuated dark-light differences. However, this is unlikely because the stress responses caused by blood sampling, if any, should affect CORT levels equally in both dark and light conditions, and thus would not severely attenuate dark-light differences. In addition, our blood sampling procedure, which was completed within 3 min, is considered appropriate for measuring the baseline levels of blood CORT concentrations (Wada et al., 2008).

The neurophysiological mechanisms by which song production is regulated by intrinsic motivation, ambient light, and environmental stress remain unclear and should be investigated in further studies. Our results in ZFs that LO suppresses song production but enhances intrinsic singing motivation strongly suggest that darkness affects only the song initiation process, but not intrinsic singing motivation, which normally drives song initiation in light conditions. In addition, the enhancement of intrinsic singing motivation depending on the duration of LO periods suggests that intrinsic singing motivation spontaneously and gradually increases over time while song production is suppressed, but substantially decreases after the song is produced. Moreover, unlike darkness, strong environmental stresses (such as predation risk) appear to suppress song production, presumably by decreasing intrinsic singing motivation. Because modulations of intrinsically motivated behaviors by ambient light and environmental stresses are universal phenomena observed not only in songbirds but also in many other bird and animal species, understanding the neurophysiological mechanisms of how undirected singing is regulated by intrinsic motivation and modulated by ambient light and environmental stress would provide great insights into the physiology of animal behavior and cognition.

## Data Availability

The raw data supporting the conclusion of this article will be made available by the authors, without undue reservation.
